# Research of DOA Estimation Based on Single MEMS Vector Hydrophone

**DOI:** 10.3390/s90906823

**Published:** 2009-08-31

**Authors:** Wen Dong Zhang, Ling Gang Guan, Guo Jun Zhang, Chen Yang Xue, Kai Rui Zhang, Jian Ping Wang

**Affiliations:** 1 National Key Laboratory For Electronic Measurement Technology, North University of China, Taiyuan 030051, China; E-Mails: wdzhang@nuc.edu.cn (W.D.Z.); zhangguojun1977@nuc.edu.cn(G.J.Z.); xuechenyang@nuc.edu.cn (C.Y.X.); 2 Key Laboratory of Instrument Science and Dynamic Measurement (North University of China) of Ministry of Education of China, Taiyuan 030051, China; E-Mails: zbdxzkr2006@163.com (K.R.Z).; wangjianping119@126.com (J.P.W.)

**Keywords:** MEMS, piezoresistive effect, vector hydrophone, DOA estimation, beam-forming

## Abstract

The MEMS vector hydrophone is a novel acoustic sensor with a “four-beam-cilia” structure. Based on the MEMS vector hydrophone with this structure, the paper studies the method of estimated direction of arrival (DOA). According to various research papers, many algorithms can be applied to vector hydrophones. The beam-forming approach and bar graph approach are described in detail. Laboratory tests by means of the a standing-wave tube are performed to validate the theoretical results. Both the theoretical analysis and the results of tests prove that the proposed MEMS vector hydrophone possesses the desired directional function.

## 1. Introduction

Vector hydrophones can obtain both the acoustic pressure and acoustic particle velocity simultaneously, and therefore can gather more information about a point in the sound field than an ordinary acoustic pressure hydrophones. Thus, they have a unique advantage and wide applicability [1–4].

As early as in the 1940s, the United States developed a sound pressure gradient vector hydrophone, and in 1970s, the vector hydrophone was successfully applied in the DIFAR sonobuoy system. In the development of vector hydrophones, the United States and Russia have taken the lead in the World, and vector hydrophones with stable performance have not only gone into the engineering phase [5–6], but explorations in the field of vector hydrophone calibration have also been carried out [7]. In the field of the vector hydrophone signal processing, Nehorai established a position measurement model with a combination of sensor arrays in 1994, and gave two methods to measure the orientation error, the methods are the Cramer-Rao lower limit and the use of a single combination of sensors, respectively [8]. Because traditional hydrophones are piezoelectric ceramics, their structure and principle are also relatively simple, so the United States and Russia also typically use the resonant vector hydrophone based on the piezoelectric accelerometer.

The MEMS vector hydrophone is designed based on the piezoresistive effect of silicon, The beam microstructure is manufactured by means of a silicon-on-insulator (SOI) wafer with MEMS technology. Compared with traditional piezoelectric hydrophones, the MEMS vector hydrophone has the characteristic of a being a single sensor with directional functions. In theory, two sensors can locate the target.

## 2. MEMS Vector Hydrophone

**Figure 1.**
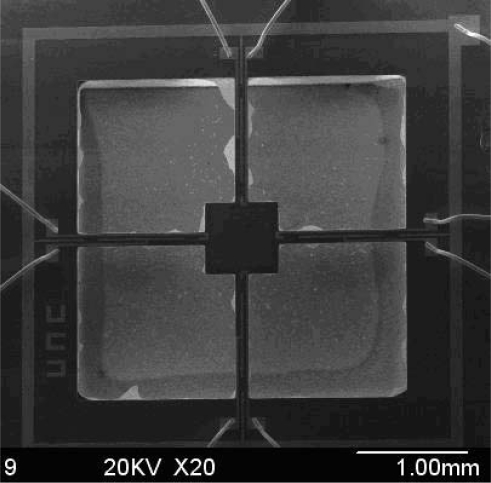
SEM images of the microstructure.

In this paper, we will discuss a novel hydrophone which is developed from a typical accelerometer structure. The SEM image (top view) of the microstructure is shown in Figure 1.

**Figure 2.**
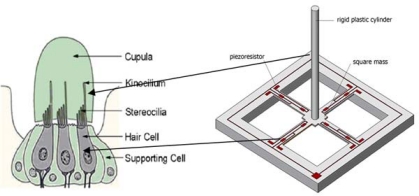
Schematic view of the bionic structure of the MEMS hydrophone.

**Figure 3.**
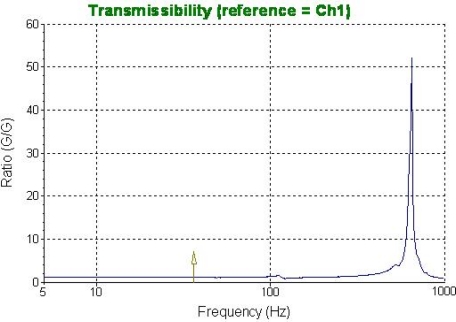
Frequency response of the structure.

As we can see in this Figure, it is a typical mass-beam accelerometer structure, which is based on the piezoresistive effect. In order to improve the sensitivity, a cylinder (diameter: 200 μm, length: 7,000 μm) was glued in the center of the mass [9]. Figure 2 shows the bionic microstructure of MEMS vector hydrophone, which consists of two parts: four high-precision cantilever beams and a rigid cylinder which is fixed at the central of the square mass. Figure 3 shows the frequency response of this geometry from 0 Hz to 1 KHz, BK 8305 as a standard accelerometer (sensitivity is 60 mV/g), the sensitivity of this structure in the X direction is 0.7552 mv/g.

According to the auditory principle of a fish’s lateral line, we use a rigid cylinder as a stereocilia which can improve the sensitivity. When the sound signal is sensed by the cylinder, the piezoresistors located at the beam transform the sound signal into strain and finally into a differential output voltage signal via the Wheatstone bridge circuit. So the sound signal can be detected.

## 3. The Principle of Direction

The sound field is the vector field, and a plane wave is a longitudinal wave, so the vector hydrophone measures the direction of sound vibration (vibration velocity v̄) and sound intensity flow Pv̄ (where P is acoustic pressure) is the direction of sound propagation, which is the goal orientation, so the measurement Pv̄ can estimate the DOA.

From the acoustic theory, we can see the plane wave acoustic pressure can be expressed as [10–12]:

(1) $$p(r,\;t)=p_{0}\;e^{j(\vec{k}\vec{r}-\mathrm{wt})}=p_{0}\;\text{exp}[j(\mathrm{kx}\;\text{cos}\;\alpha \;\text{cos}\;\theta +\mathrm{ky}\;\text{cos}\;\alpha \;\text{sin}\;\theta +\mathrm{kz}\;\text{sin}\;\alpha -\mathrm{wt})]$$

**Figure 4.**
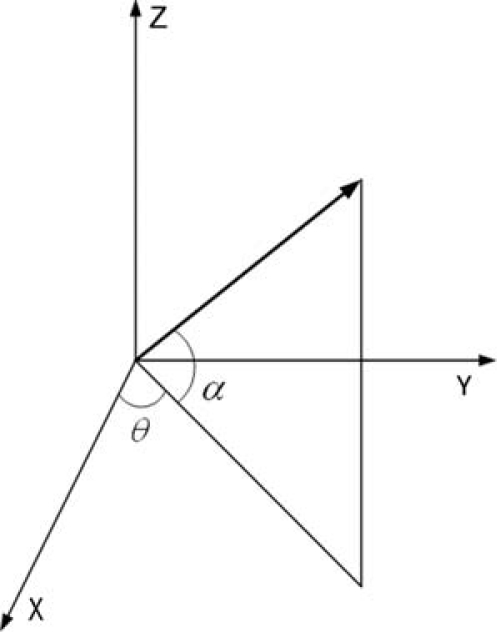
Wave vector’s projection in the coordinate system.

Where *k* is a wave vector, indicating the direction of sound propagation, *α* is the angle between *k* and horizontal plane, *α* ∈ [−π/2, π/2], *θ* is the angle between the projection in the horizontal plane of *k* and x-axis, *θ* ∈ [0,2π], As shown in Figure 4:

In homogeneous medium, the sound field equation of motion is:

(2) $$\frac{\partial \vec{v}}{\partial t}+\frac{1}{\rho_{0}}\nabla p=0$$

Substituting this into Expression 1,we get:

(3) $$\bar{v}=\frac{p_{0}}{\rho_{0}c}\;\left(\text{cos}\theta \text{cos}\alpha \vec{\xi }+\text{sin}\theta \text{cos}\alpha \vec{\eta }+\text{sin}\alpha \vec{\tau }\right)$$

Where *ξ, η, τ* is the unit vector of x, y, z axis, *ρ*_0_ is the medium density, *c* is the velocity of sound in the medium. Equation (3) shows that the ratio between acoustic pressure and the three components of particle velocity is just a constant, their wave is the same, so pressure and velocity of the plane wave are totally relevant.

From (3) we can get the three components of the particle velocity:

(4) $$\begin{array}{l} v_{x}=\frac{p}{\rho_{0}c}\;\text{cos}\;\theta \text{cos}\;\alpha \\ v_{y}=\frac{p}{\rho_{0}c}\;\text{sin}\;\theta \;\text{cos}\alpha \\ v_{z}=\frac{p}{\rho_{0}c}\;\text{sin}\;\alpha \end{array}$$

and:

(5) $$\theta =\text{arctan}\left(\frac{v_{y}}{v_{x}}\right)$$

(6) $$\alpha =\text{arctan}\left(\frac{v_{z}}{\sqrt{v_{x}^{2}+v_{y}^{2}}}\right)$$

So, as long as we measured the two components ν_x_, ν_y_ of particle velocity in the horizontal plane, we can get the azimuth *θ* of the sound source in the horizontal plane from Equation (5), three velocity components can get the pitch angle *α* from Equation (6). These are the basic principles that the vector hydrophone uses to determine a sound source’s position.

## 4. Two Approaches to DOA Estimation

According to the different types of noise background and signals, there are several approaches to estimate azimuth angle based on a single vector hydrophone [13], such as the average acoustic power approach, line-spectra estimation, the bar graph approach and the beam-forming approach. However, the first two methods can’t do well in the condition of either line-spectra coherent interference lying in broad band signal or broadband coherent interference lying in line-spectra signal. In this paper, we apply the beam-forming approach and bar graph approach to the DOA estimation based on a single MEMS vector hydrophone.

### 4.1. Beamforming Approach

The term beam-forming refers to the various output signals whereby a vector hydrophone can determine space directivity after processing (weighted, delay, summation etc.), It can focus the receive direction of hydrophone on a direction, which is equivalent to a beam, rotating the beam to find the maximum peak, that is the target azimuth. The algorithm can be seen as a space filter that can separate the signal from interference, according to the azimuth difference between signal and interference.

There are many weighted modes, this paper only analyzes two of them. If only in the plane, according to (4) we can obtain the following equation:

(7) $$\begin{array}{l} v_{x}=\frac{p}{\rho_{0}c}\;\text{cos}\;\theta \\ v_{y}=\frac{p}{\rho_{0}c}\;\text{sin}\;\theta \end{array}$$

The first weighted method: *v_x_*(*t*) cos *ψ* + *v_y_*(*t*) sin *ψ*

Substituting (7) into it and assuming $\frac{1}{\rho_{0}c}=1$, we have the following equation:

(8) $$v_{x}\;(t)\;\text{cos}\;\psi +v_{y}\;(t)\;\text{sin}\;\psi =p\times \text{cos}(\theta -\psi )$$

The second weighted method, adds the acoustic pressure *p*(t), so we get:

(9) $$p+v_{x}\;(t)\;\text{cos}\;\psi +v_{y}\;(t)\;\text{sin}\;\psi =p(1+\text{cos}(\theta -\psi ))$$

**Figure 5.**
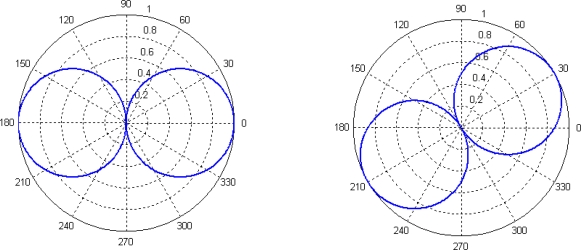
Directivity patterns of function (8) (*θ* = 0° and *θ* = 30°).

**Figure 6.**
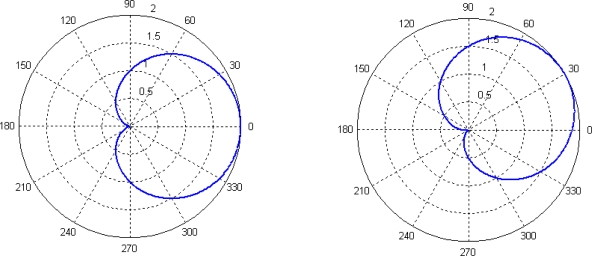
Directivity patterns of function (9) (*θ* = 0° and *θ* = 30°).

The directivity patterns of two weighted functions (8) and (9) are as follows, respectively:

The Figure 5 is “8”-shaped with bilateral directivity, which can induce port and starboard ambiguity, however, the Figure 6 is heart-shaped with unilateral directivity. Therefore, we use the second weighted form to reduce the side-lobe. If we don’t add *p*(t), the outcome will have two peaks.

**Figure 7.**
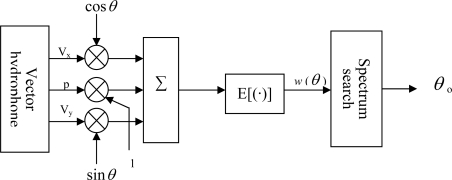
Principle chart of the beam-forming approach.

The principle chart of the beam-forming approach is shown in Figure 7, weighting the three way signal *p*(t), ν_x_(t) and ν_y_(t) output of hydrophone by 1, cos*θ*, sin*θ* and summing them. The result is:

(10) $$y(t)=p(t)+v_{x}\;(t)\;\text{cos}\;\theta +v_{y}\;(t)\;\text{sin}\;\theta$$

The average power of *y* (t) is:

(11) $$w(\theta )=E\left[\left|y{\left(t\right)}^{2}\right|\right]$$

where, *E*[ ] – ensemble average; *w*(*θ*) – space spectrum of the output, is a function of *θ*, reflecting the space distribution of energy. Search the max of *w*(*θ*) in *θ* ∈ [0,2π], *w*(*θ*) can acquire the max, only when *θ* = *θ*_0_, so *θ*_0_ is the azimuth angle of the sound signal. Virtually, this is a process of beam-forming [14].

### 4.2. Bar Graph Approach

From Equation (5) we know that, by simply calculating the three velocity components measured by a vector hydrophone, we can identify the azimuth angle *θ* and pitch angle *α* of a sound source. By determining the positive and negative of ν_x_ and ν_y_, a single vector hydrophone can estimate the direction of the sound source. Although this method is simple, the error would be great, this is mainly because the three velocity components ν_x_, ν_y_and ν_z,_ measured by the vector hydrophone not only contain a valid signal, but also noise. Noise has a great impact on the estimated results, and this impact is more serious with lower SNR (signal-to-noise ratio).

We assume the signals received from vector hydrophone are as follows:

(12) $$p(t)=p_{s}\;(t)+p_{n}\;(t)$$

(13) $$v(t)=v_{s}\;(t)+v_{n}\;(t)$$

In the equation,

*p_s_*(*t*)- acoustic pressure generate by target sound source;
ν_s_(*t*)- particle velocity generate by target sound source;
*p_n_*(*t*)- acoustic pressure of isotropic noise field;
ν*_n_*(*t*)- particle velocity of isotropic noise field.

Target signal and interference noise is independent of each other, and they are ergodic. Calculating average sound intensity by above formula, we get:

(14) $$\bar{I(t)}=\bar{p(t)\cdot v(t)}=\bar{\left(p_{s}\;(t)+p_{n}\;(t))\cdot (v_{s}\;(t)+v_{n}\;(t)\right)}\approx \bar{p_{s}\;(t)v_{s}\;(t)}$$

For *p_n_*(*t*), ν*_n_*(*t*) and *p_s_*(*t*) are independent of each other, the value of the average cross-multiplied is small, which can be neglected.

(15) $$\theta =\text{arctan}\left[\frac{\bar{I_{y}\;(t)}}{\bar{I_{x}\;(t)}}\right]$$

**Figure 8.**
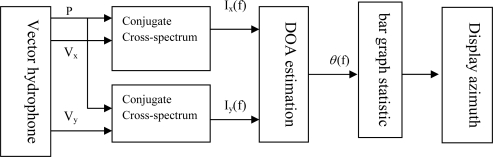
Principle chart of the bar graph approach.

Bar graph estimation is a statistical approach based on cross-spectrum estimation. First we calculate the angle corresponding to each frequency, and then count the probability density of each estimated value, to get the estimated curve of a certain moment, the curve maximum position corresponds to the estimated value of which is the target position. Figure 8 shows its principle chart [13], acoustic pressure and particle velocity are calculated by the conjugate and cross-spectrum. If *P*(*f*), *V_x_*(*f*) and *V_y_*(*f*) correspond to the FFT of the acoustic pressure and particle velocity signal *p*(t), ν_x_(t) and ν_y_(t), we can get the expression of calculated azimuth:

(16) $$\theta =\text{arctan}\left[\frac{\bar{I_{y}\;(t)}}{\bar{I_{x}\;(t)}}\right]=\text{arctan}\left(\frac{R\left[P*(f)V_{y}\;(f)\right]}{R\left[P*(f)V_{x}\;(f)\right]}\right)$$

where, *R*[ ] – get real component; “*” – conjugate.

Bar graph statistics mainly estimates the azimuth of every frequency, if 1° is the statistical unit, we get:

(17) k=[θ(f)×180/π]

(18) φ(k)=φ(k)+1

In Formula (17), [ ] – get integer. *θ*(*f*) is the azimuth value of certain frequence, transforming *θ*(*f*) to angle *k*. *φ* is an array, which stores the frequence of every angle in [−180°,180°], initialization of the array is zero, when we get a value of k, the frequence *φ*(*k*) of corresponding to *k* add one in array. The statistical result is the azimuth estimated value.

## 5. Test and Results

**Figure 9.**
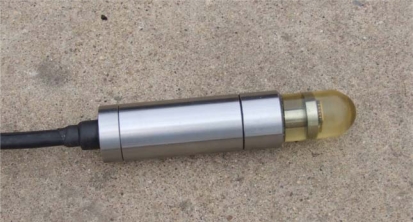
The photo of the MEMS vector hydrophone.

**Figure 10.**
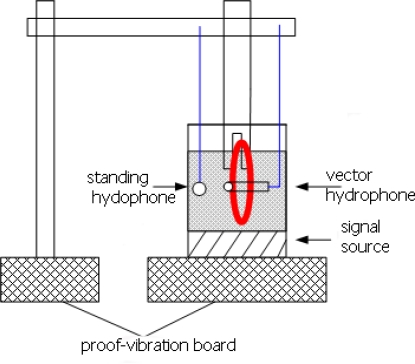
Principle chart of the calibration instrument.

The DOA estimation experiment has been completed by means of the a test vector hydrophone instrument in the laboratory, Figure 9 shows the photo of MEMS vector hydrophone being tested. Its receiving sensitivity is up to −160 dB(0dB = 1 V/μpa). Figure 10 shows the principle chart of the test instrument, the instrument can emit standing wave in a circular tube. The sound source is located in the tube’s bottom, so a uniform and stable sound field is built in the tube. We can change the angle of wave incidence by adjusting hydrophone’s position. The computer collects the sound information transformed by hydrophone, and processes the data, and as a result, the direction of the sound signal can be estimated.

## 5.1. Directed to the Single-frequency Signal

**Figure 11.**
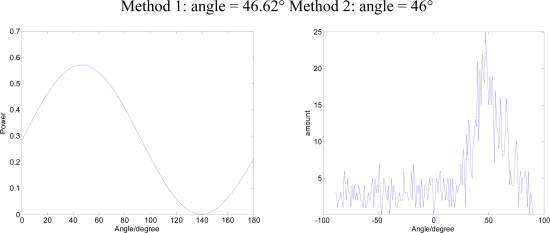
The results of the two methods (DOA = 45°).

Using a signal generator to generate a single-frequency sine wave S_1_(t), DOA is 45°. The Data Acquisition Card collects a three-way signal at the same time P, V_x_, V_y_, data sampling rate is set to 10 KHz. The two methods are applied to DOA estimates. The results in Figure 11 show Method 1 for the beam-forming approach, Method 2 for the bar graph approach.

### 5.2. Directed to the Noise Signal

**Figure 12.**
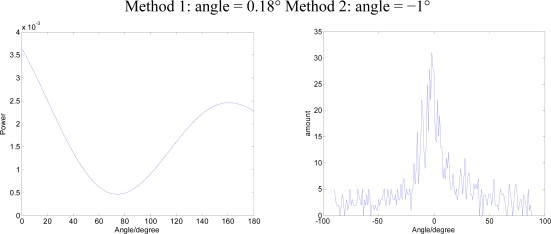
The results of the two methods (DOA=0°)

As sound source a broadband signal (white noise) S_2_(t) is used, DOA is 0°. The results of the two methods are shown in Figure 12:

### 5.3. Directed to the Line-Spectrum Signal in the Context of Noise

**Figure 13.**
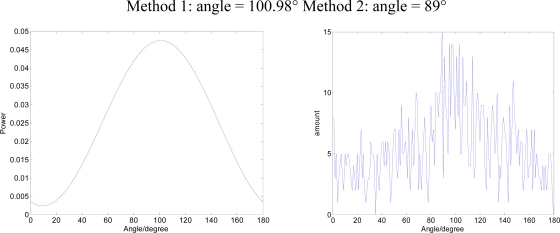
The results of the two methods (DOA = 90°).

As sound source we enter a single-frequency signal with attached white noise S_1_(t) + S_2_(t), DOA is 90°. The results of the two methods are shown in Figure 13:

## 6. Conclusions

In this paper, by testing and analyzing the directional functions of a MEMS vector hydrophone in an ideal room environment, we obtained better results. From the test results, the MEMS hydrophone can not only direct but also have high orientation accuracy. For a continuous wave, two methods are feasible, but because of the requirements of frequency resolution, the bar graph approach is not suitable for pulse waves. There are two main reasons of error: first, because of the inaccuracy of the measured angle and the other is the rounding error of the bar graph. It remains to be determined whether the MEMS vector hydrophone can achieve good goal orientation or not in the actual marine environment.
